# Optimising the choice of normalisation method for use in machine-learning classification of human blood plasma ambient ionisation mass spectra

**DOI:** 10.1016/j.ijms.2025.117553

**Published:** 2026-02

**Authors:** Annabel S.J. Eardley-Brunt, Liwen Song, The Oxford Acute Myocardial Infarction Study, The Oxford Abdominal Aortic Aneurysm Study, Claire Vallance

**Affiliations:** a Department of Chemistry, University of Oxford, Chemistry Research Laboratory, 12 Mansfield Rd, Oxford OX1 3TA, UK; b Division of Cardiovascular Medicine, Radcliffe Department of Medicine, University of Oxford, John Radcliffe Hospital, Oxford, OX3 9DU, UK; c Nuffield Department of Surgical Sciences, University of Oxford, John Radcliffe Hospital, Oxford, OX3 9DU, UK

**Keywords:** Data normalisation, Preprocessing, Multivariate analysis, Machine learning, Clustering ratio, Atmospheric solids analysis probe mass, spectrometry (ASAP-MS)

## Abstract

Construction of large mass spectrometric data sets usually involves some combination of normalisation, scaling, and transformation of individual mass spectra in order to correct for technical (and sometimes biological) variation. Many different approaches to data normalisation have been reported, and there is no particular consensus on the best approach. The present study systematically evaluates a set of 24 normalisation, scaling, and transformation methods, and their 420 possible combinations, in the context of atmospheric solids analysis probe (ASAP) mass spectra of human blood plasma. The plasma samples came from two separate cohorts of patients, enrolled respectively in the Oxford Acute Myocardial Infarction (OxAMI) and Oxford Abdominal Aortic Aneurysm (OxAAA) clinical studies. Within each cohort, patients are classified according to a number of different clinical variables. We have investigated the effect of normalisation, scaling, and transformation method on subsequent clustering of the data into the classes of interest, and on machine-learning based classification of the data into the categories of interest. The choice of method was found to have a substantial effect on data clustering, measured via the clustering ratio *C*_R_, but a much smaller effect on machine-learning based classification, quantified via Cohen’s *κ* statistic. New intensity-histogram-based normalisation methods were found to have the greatest effect on clustering, while mean, median, vector, and AUC normalisation yielded the best machine-learning classification performance across multiple algorithms. High clustering ratios do not necessarily correlate with improved supervised classification outcomes, underscoring the need to consider subsequent data analysis methodology carefully when optimising data preprocessing pipelines.

## 1 Introduction

Atmospheric solids analysis probe mass spectrometry (ASAP-MS) is a form of ambient ionisation mass spectrometry (AIMS) capable of rapid analysis of solid and liquid samples in their native state [1]. Within the ion source, a flow of hot N_2_ gas desorbs analytes from the surface of a glass capillary, and an electric discharge leads to ionisation via various gas-phase ion-molecule reactions, resulting mainly in proton transfer to the analyte. The extent of fragmentation following ionisation can be controlled to some extent by the extraction potentials employed to transfer the ions into the mass analyser, which is usually a single quadrupole in benchtop instruments, and a time-of-flight analyser in larger, more sophisticated instruments. With either type of analyser, the result is an untargeted mass spectrum of the sample. A key appeal of ASAP-MS is its simplicity and ease of use, enabling rapid sample characterisation without the need for time-consuming and costly sample preparation such as separation, extraction and chromatography [2].

As a result of these characteristics, ASAP-MS holds considerable potential for applications in clinical settings, e.g. for rapid tissue typing of biopsy samples, or for metabolic and lipid fingerprinting in a variety of contexts. Combining the latter with machine learning or other pattern-recognition approaches opens up the intriguing possibility of a quick mass spectrometric blood test with the ability to deliver diagnostic and/or prognostic information on a range of diseases, complementing a variety other approaches currently in development [3–6]. Recently, we have been investigating such an approach in the context of cardio-vascular diseases (ST-elevated myocardial infarction or ‘STEMI’, and abdominal aortic aneurysm (AAA or ‘triple A’)), determining whether ASAP mass spectra of blood plasma can be used to predict outcomes such as the extent of cardiac damage and the likelihood of future complications, in the case of STEMI, and aneurysm presence, size, and growth rate in the case of AAA [7].

As a result of the manual sample introduction step, the experimental uncertainties inherent in ASAP-MS measurements tend to be somewhat higher than in other types of mass spectrometry [8]. To make a measurement, the probe tip is touched onto or dipped into the sample and then inserted into the spectrometer ion source. The simplicity of the sampling process is a great strength of the technique, but makes it difficult to control the amount of sample introduced with a high degree of precision. As a result, absolute quantitation between repeat measurements is unfeasible without the use of internal concentration standards (i.e. sample ‘spiking’, which is not well suited to the applications under consideration) or normalisation techniques. Data normalisation can in principle mitigate the effects of measurement-to-measurement variation in the amount of sample introduced into the ion source, enabling comparison of peak intensities between measurements.

### 1.1 Normalisation of mass spectrometry data

For MS-based -omics research, a variety of pre-processing approaches have been investigated for improving the biological information content and reducing the impact of error and noise in a data set [9]. These can be classified into *normalisation, scaling*, and *transformation* methods. The definitions of these categories are often somewhat ambiguous and overlapping, but in the present work, they will be defined as follows.

- **Normalisation methods:** Normalisation is an operation carried out on each mass spectrum individually in order to make the set of spectra comparable in terms of total signal or relative intensities. Common normalisation methods include scaling each mass spectrum so that it has unit area under the curve, or dividing each mass spectrum by the intensity of the most intense peak, so that all peak intensities lie in the range [0, 1]. Normalisation methods mitigate differences in total ion count between measurements, making it easier to identify or compare patterns in peak ratios across measurements. Details of the normalisation methods used in the present study will be given in Section 2.1.1.
- **Scaling methods:** Rather than considering each mass spectrum individually, scaling methods (also known as ‘standardisation’ methods) essentially normalise each *m*/*z* bin over the full set of mass spectra, with the aim being to make each *m*/*z* bin comparable across the set of spectra in terms of variance and magnitude. An example is mean centre scaling, in which the mean values of the intensity for each *m*/*z* across all mass spectra are determined, and the intensities for each *m*/*z* value in the individual spectra are divided by the corresponding mean value. Details of the scaling methods used in the present work will be given in Section 2.1.2.
- **Transformation methods:** These apply a function to the peak intensities, changing the intensity distribution (and therefore the absolute and relative peak intensities) within the mass spectra. For example, taking the log of the peak intensities reduces the dominance of large *m*/*z* peaks and spreads out the intensities of smaller ones. Details of the transformation methods used in the present work will be given in Section 2.1.3.

A literature review led us to conclude that there is no ‘gold standard’ normalisation method, with recommendations that researchers should assess a range of methods to determine which best suits their data [10–14]. A literature search using the *Web of Science* search tool (Clarivate Web of Science, Copyright Clarivate 2023), including papers published from 2013 to 2023 that featured ‘atmospheric solids analysis probe mass spectrometry’ yielded 33 papers described as related to the fields of analytical chemistry or biomedical research, 19 of which featured some form of data normalisation. Of these, 13 used relative abundance or unit mass normalisation methods (e.g. [15]), two used internal calibration peaks (e.g. [16]), three used a combination of Pareto scaling and relative abundance (e.g. [17]), and one used a combination of quantile normalisation, log transformation, and Pareto scaling [18]. These named methods will be defined in the next section. This showed that even within the small but growing field of ASAP-MS, a wide range of normalisation methods are in use.

**Fig. 1:**
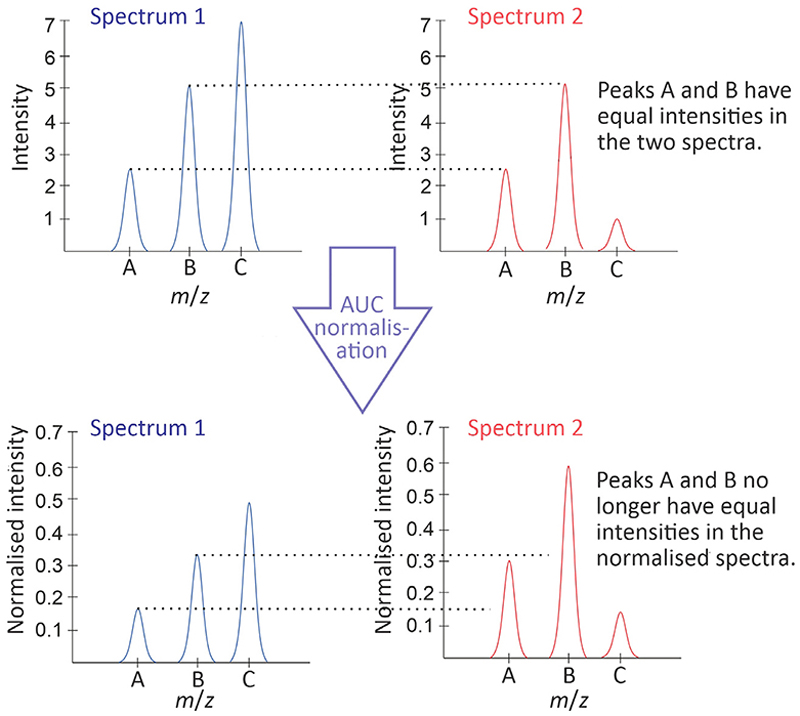
An illustration of the impact of AUC normalisation on the absolute and relative intensities of three *m*/*z* peaks (labelled A, B, C) in two different spectra.

One of the challenges presented by -omics data is that metabolites and biomolecules do not self-average, meaning that a concentration increase in one group of metabolites is not generally balanced by a decrease in another group [11]. Some approaches to normalisation, such as unit AUC and mean/median methods, do not account for this behaviour, and may lead to artifacts in the normalised data. An example is shown in Fig. 1. In the unnormalised spectra, the peak labelled C is the most intense peak in spectrum 1, but has a much lower intensity in spectrum 2, and the peaks labelled A and B have identical intensities in the two spectra. However, using area-under-the-curve normalisation, the high intensity of peak C leads to suppression of the intensities for peaks A and B in Spectrum 1, yielding very different normalised intensities for these peaks in the two normalised spectra.

Normalisation, scaling and transformation methods can be combined in different ways to produce composite methods that reduce the impact of variation in a data set in an optimal way, which the optimum choice of method depending on the analysis to be conducted. Open-source software packages such as Metaboanalyst provide a platform on which users can compare the effects of various normalisation methods on data sets, before performing multivariate statistics analysis with the goal of classifying the data into two or more categories [19,20]. However, the software currently offers no way to quantify the impact of different methods other than a trial-and-error approach based on visual inspection of the results. The optimum normalisation approach for a particular data set can in fact be assessed using a number of methods, including statistical methods, unsupervised clustering analysis such as principal component analysis (PCA) [21], and supervised ML methods such as K-nearest neighbours(KNN) classification [22]. Using a number of ASAP-MS data sets collected for complex biological samples, we have compared the effect of a large number of normalisation methods by assessing the ability of machine learning models to classify the resulting processed mass spectra into the appropriate categories.

### 1.2 Sources of error and uncertainty in ASAP-MS data

As noted earlier, normalisation, scaling and transformation methods are commonly employed to reduce the impact of measurement uncertainty on a biological data set and to enable inter-sample comparisons. It is important to understand sources of error and uncertainty in the measurements as fully as possible in order to ensure that appropriate techniques are used to minimise these at the experimental design stage, the experimental measurement stage, and finally the data processing stages. Van den Berg et al. [9] describe this process well in the context of variation within biological MS data. Sources of uncertainty in our ASAP-MS measurements on blood plasma can be categorised into sample-related variation and technical variation, with the latter including both variation caused by sample loading, and instrument-related variation.

**Sample-related variation:** Natural variation in the concentrations of common, abundant metabolites across different individuals can be considerable, with the most abundant metabolites not necessarily of interest in the context of a particular disease [9]. This is a common problem in untargeted metabolomics studies, as abundant molecules can obscure the measurement of less abundant species. One also needs to consider both ‘induced’ and ‘uninduced’ sources of biological variation. Induced biological variation describes how the biological context of each metabolite influences the amount of natural variation observed [23]. For example, primary metabolites linked to central metabolic processes tend to be relatively constant, but metabolites related to peripheral and secondary processes may show much larger variation [24]. In contrast, uninduced biological variation occurs when a metabolite present at the same concentration in different samples does not yield the same signal in a measurement, often showing large variations even under the same experimental conditions. The reasons for this are not well understood, but may arise from differences in the physical and chemical properties of the biological matrix, such as viscosity, fat content, and water content.

**Technical variation:** Technical variation in ASAP-MS can arise from varying amounts of sample being loaded onto the ASAP probe, and from other factors such as fluctuations in the carrier gas pressure and temperature and the environment of the laboratory, including temperature and humidity [25]. As some of these physical factors vary on an hourly to daily basis, they may manifest as batch effects, whereby spectra are observed to change between batches of measurements, introducing unpredictable batch-dependent structure into the data [26, 27]. Batch effects can have a large impact on atmospheric pressure ionisation mass spectrometry (AIMS) measurements, and need to be carefully considered during both experimental setup and data analysis. Batch-correction algorithms offer a potential method of reducing the impact of batch effects, but can also obscure patterns in the data set [28]. Alternatively, batch effects can be mitigated in many cases by ensuring that samples are measured randomly over a large number of batches, by using control samples, and by employing standard normalisation techniques [26,27,29]. As mentioned above, in complex biological samples, molecules that are highly abundant can obscure the measurement of less concentrated molecules; for example, the presence of some ions can suppress ionisation of other species, leading to reduced detection sensitivity. Finally, it is worth being aware that the commonly made assumption of errors being symmetric or normally distributed is rarely true. Using algorithms that assume normal distributions can impose structured (‘heteroscedastic’) error on a data set if the assumption of normally distributed data is incorrect [9,30].

## 2 Materials and methods

### 2.1 Normalisation, scaling and transformation methods

A total of 24 normalisation, scaling, and transformation methods, listed in Table 1 and defined further in the next section, were evaluated in relation to ASAP-MS data. The methods were applied in various combinations, sequentially in the order (i) normalisation, (ii) scaling and (iii) transformation. This choice was made in order to first reduce variation between repeats, then to reduce inter-sample variation within features, before transforming the data distributions. This generated 420 different method combinations, including a blank method in which no normalisation, scaling or transformation was performed.

In the equations in the following section, *I*_*m*,*S*_ and *N*_*m*,*S*_ are, respectively, the unnormalised and normalised intensities in mass bin *m* of spectrum *S*.

#### 2.1.1 Normalisation methods

**AUC normalisation:** The intensity *I*_*m*,*S*_ in each *m*/*z* bin is divided by the sum (or integral) Σ_*m*_ *I*_*m*,*S*_ of the intensities over all mass bins.

(1) $$N_{m,S}=\frac{I_{m,S}}{\Sigma_{m}I_{m,S}}$$

**Mean and median normalisations:** The intensity *I*_*m*,*S*_ in each *m*/*z* bin is divided by the mean value, *Ī_S_*, or the median value, *Ĩ_S_*, of all the *m*/*z* bin intensities, respectively.

(2) $$N_{m,S}=\frac{I_{m,S}}{{\bar{I}}_{S}}$$

(3) $$N_{m,S}=\frac{I_{m,S}}{{\tilde{I}}_{S}}$$

**Vector normalisation:** The intensity *I*_*m*,*S*_ in each *m*/*z* bin is divided by the square root of the sum of the squared peak intensities.

(4) $$N_{m,s}=\frac{I_{m,S}}{\sqrt{\Sigma_{m}I_{m,S}^{2}}}$$

**Minimised vector normalisation:** Each spectrum is treated as a vector in *N*-dimensional space, with *N* equal to the number of *m*/*z* bins. The spectra are then normalised based on their deviations from the centroid vector (mean vector) of all spectra, **Ī_S_**. The deviation from the centroid, defined by **Ī_S_** − *k*_*S*_ **I**_**S**_ is then minimised with respect to *k*_*S*_, with the normalised spectrum given by

(5) $$N_{m,S}=k_{S,\text{min}}I_{S}$$

**Quantile normalisation:** This normalisation approach forces all samples in a data set to have the same overall distribution of intensities. The mass spectra are considered as a list of intensity values, and the normalisation process proceeds as follows [31,32]:

1. Sort the intensities within each spectrum from lowest to highest.
2. Average the sorted intensity values across all spectra at each rank.
3. Replace the original sorted intensity values in each spectrum with the averaged values.
4. Unsort the data, i.e. restore the original order of peaks for each spectrum.

**Histogram distribution normalisation:** Histogram normalisation was developed specifically for the purposes of the present study in order explore the idea of normalising the spectra to the area under the curve of a peak height distribution, represented as a histogram of peak intensities. In this way the normalisation constant is minimally affected by a few peaks with wildly varying intensities, and is mostly determined by the intensities of peaks with fairly reproducible intensities.

**Fig. 2:**
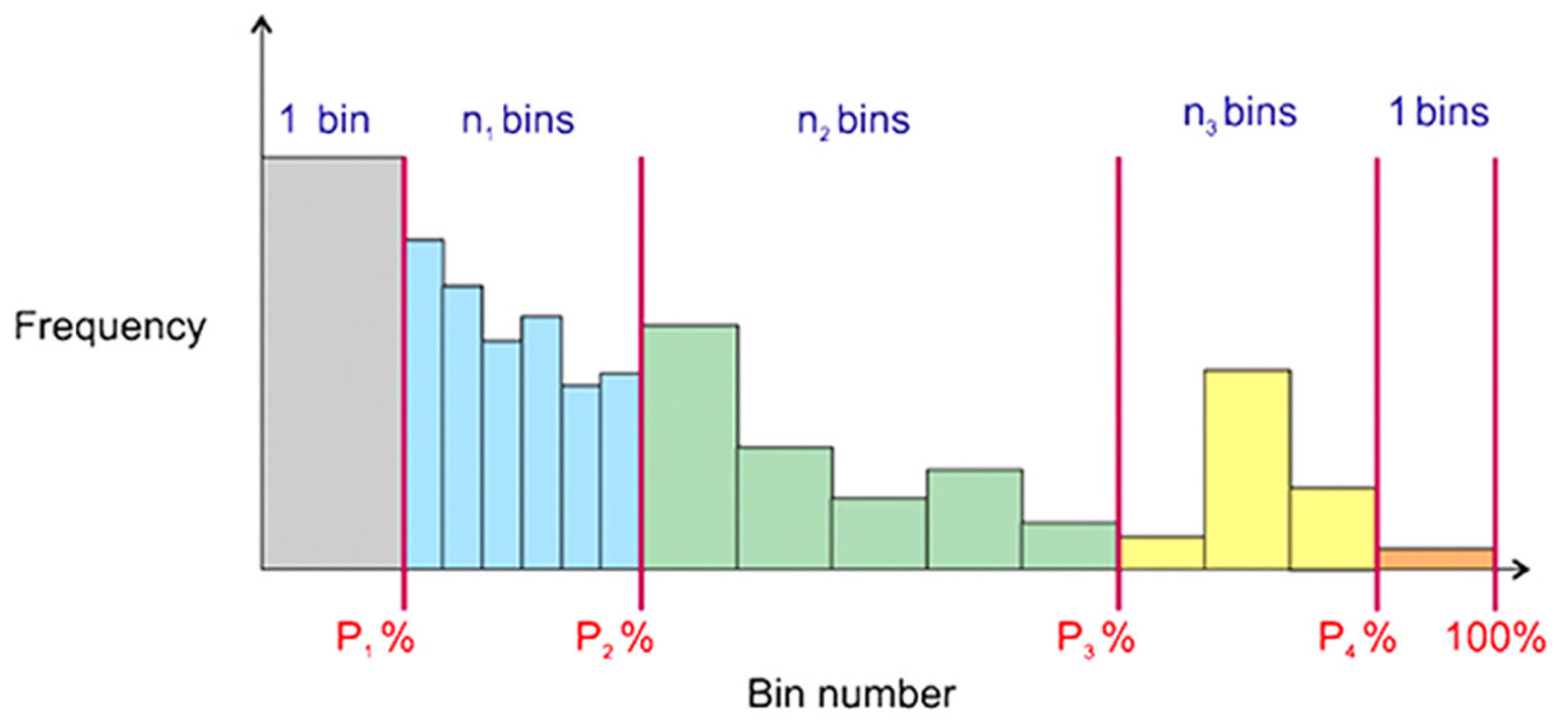
The distribution of histogram bins used in the intensity histogram normalisation methods. Bin segments are shown separated by the red lines at locations *P*_*n*_%, with the number of bins *n* in each segment shown by the coloured bars.

The intensity histogram for each spectrum was divided into five unequal segments, as shown in Fig. 2, and the width of the bins within each segment was then optimised as follows. The choice of unevenly sized intensity bins results from the observations that (i) the peak distribution is highly skewed towards the low-mass region, with many highly variable peaks in this region, and (ii) the higher mass region is sparsely populated. The first histogram segment comprises a single bin from 0 to the lower limit percentile (*P*_1_), initially set at 20%, and represents peaks with very low intensities and background noise. The second segment, spanning intensities from *P*_1_ to *P*_2_, is given a flexible number of bins (*n*_1_) initially set at 30, and represents peaks with low intensity values. The third and fourth segments, spanning intensities from *P*_2_ to *P*_3_ and *P*_3_ to *P*_4_, respectively, also have a flexible number of bins (*n*_2_ and *n*_3_), initially set at 30 each. The final segment of the distribution is represented by a single bin and represents the most intense peaks, which often vary considerably in intensity.

The number of segments, their percentile intensity ranges *P*%, and the number *n* of bins in each segment were optimised by a genetic algorithm, using the extent to which the resulting normalised data clustered into the two categories of interest (see Section 2.5) as the goodness-of-fit function.

Three different methods were used to represent the spectra based on the final optimised histogram.

1. Histogram bin - Replace each *m*/*z* intensity with the histogram bin number into which it falls.
2. Histogram intensity - use the intensity histograms as the final representations of the mass spectra.
3. Histogram function - The normalised spectrum is the ratio of bin number to intensity for each *m*/*z*.

(6) $$N_{m,S}=\frac{\text{bin}\left[I_{m,S}\right]}{I_{m,S}}$$

#### 2.1.2 Scaling methods

**Mean and median centre scaling:** Similar to mean and median normalisation, but instead of dividing each *m*/*z* intensity by the mean or median intensity of the mass spectrum, the intensities are divided by the mean or median intensity *Ī_m_* or *Ĩ_m_* of that *m*/*z* value across all spectra in the data set.

(7) $$\begin{array}{c} N_{m,S}=\frac{I_{m,S}}{{\bar{I}}_{m}}\\ N_{m,S}=\frac{I_{m,S}}{{\tilde{I}}_{m}} \end{array}$$

**Auto scaling:** After performing mean centre scaling, each *m*/*z* intensity is then divided by the standard deviation of the intensities (before scaling) for that mass across all of the spectra. Auto scaling can decrease the importance of highly variable peaks relative to stable peaks.

(8) $$N_{m,S}=\frac{I_{m,S}/{\bar{I}}_{m}}{\sigma I_{m}}$$

**Pareto scaling:** Similar to auto scaling, but the mean-centre-scaled value is divided by the square root of the standard deviation of the initial intensity for that mass across all of the spectra.

(9) $$N_{m,S}=\frac{I_{m,S}/{\bar{I}}_{m}}{\sqrt{\sigma I_{m}}}$$

**Range scaling:** The mean-centre-scaled intensity for each *m*/*z* is divided by the range, max(*I*_*m*_)−min(*I*_*m*_), across all of the spectra.

(10) $$N_{m,S}=\frac{I_{m,S}/{\bar{I}}_{m}}{\text{max}\left(I_{m}\right)-\text{min}\left(I_{m}\right)}$$

#### 2.1.3 Transformation methods

The peak intensities *I*_*m*,*S*_ were transformed using one of the following six functions.

(11) $$N_{m,S}=\text{log}\left(I_{m,S}\right)$$

(12) $$N_{m,S}=\text{exp}\left(I_{m,S}\right)$$

(13) $$N_{m,S}={\left(I_{m,S}\right)}^{2}$$

(14) $$N_{m,S}={\left(I_{m,S}\right)}^{3}$$

(15) $$N_{m,S}={\left(I_{m,S}\right)}^{1/2}$$

(16) $$N_{m,S}={\left(I_{m,S}\right)}^{1/3}$$

### 2.2 Biological samples

The biological samples used in this study were blood plasma samples from the Oxford abdominal aortic aneurysm study (OxAAA) [33–35], and the Oxford acute myocardial infarction study (OxAMI) [36–38]. The details of these studies have been published previously. ASAP-MS measurements on samples from both patient cohorts were recorded as part of two separate studies [7,28,39] aiming to correlate features in the mass spectra with patient outcomes, with some work still to be published. With permission from the clinicians, these data were additionally used in the present project to study the impact of normalisation methods.

An AAA is the abnormal swelling of the abdominal aorta. Patients are usually monitored regularly until the aorta reaches a threshold diameter of 5 cm, at which point surgical intervention is recommended. The aim of the OxAAA study analysis was to investigate whether machine-learning algorithms could be trained to classify mass spectra according to clinical parameters such as the presence or absence of an aneurysm, and aneurysm size. For the purposes of the present study, we have included 20 samples from healthy volunteers and 20 samples from patients with small (< 5cm) aneurysms, and will investigate the effect of the various normalisation methods on the ability for ML algorithms to classify mass spectra into these two categories.

The aim of the OxAMI study analysis was to investigate correlations between the plasma mass spectra and a number of clinical parameters that quantify the extent of cardiac damage following ST-elevated myocardial infarction (STEMI, a form of severe heart attack). Every patient in the study underwent a primary percutaneous coronary intervention (pPCI, surgical intervention to relieve a blockage of the coronary artery) after suffering a STEMI. A range of clinical measurements were taken for each patient before, during and after the pPCI procedure to monitor the severity of the myocardial infarction, response to treatment, and recovery. A set of nine clinical variables were used for the classification analysis:

- **Creatinine level on admission** - Elevated creatinine levels indicate impaired kidney function, which is associated with worse outcomes and adverse effects post-STEMI. Cardiac stress commonly results in kidney damage due to left ventricular dysfunction, reducing blood flow to the kidneys [40].
- **Patient mortality** - Defined as death within 5 years of a STEMI.
- **Heart failure diagnosis (HF)** - Patient diagnosed with heart failure post-STEMI, resulting in irreversible and continuous decline in heart function.
- **Index of microcirculatory resistance (IMR)** - A measure of resistance to blood flow in the coronary micro-circulation. A high IMR measurement post-STEMI indicates poor circulation within the smallest blood vessels in the heart muscle, and is associated with worse outcomes. [41].
- **MDRD equation estimated glomerular filtration rate (MDRD**
- **eFGR)** - A further estimation of kidney function based on creatinine levels and patient characteristics [42].
- **Microvascular obstruction (MVO)** - The persistence of blockage or poor vascularisation of the microvasculature in the heart muscle despite successful restoration of flow. MVO is measured post pPCI via a cardiac MRI scan, with non-zero values indicating permanent damage or loss of heart function [43].
- **Ischaemic time (ITime)** - The time between the onset of STEMI symptoms and pPCI intervention [44].
- **Thrombus score (TScore)** - A visual measure of thrombus burden (size of the thrombus in relation to the size of the blood vessel) graded from 0–5, with 0 indicating no thrombus and 5 indicating a total occlusion. The majority of STEMI patients will have a TScore of 4 or 5 [45].
- **Peak troponin measurement** - The maximum level of cardiac troponin, a protein indicative of cardiac trauma, measured via a blood test on hospital admission [46].

Subsets of the complete set of mass spectra recorded for each study were chosen for a series of binary classification analyses. The categories used for this classification, together with the number of patient samples/mass spectra per class for each clinical variable, are listed in Table 2.

Previous analysis on the OxAMI data using simple area-under-the-curve normalisation during the data preprocessing had yielded weak to moderate success at classification [39], and it was hypothesised that improvements may be achieved through optimisation of the normalisation method. Previous analysis on the OxAAA data had yielded moderate to strong success at classification when employing AUC normalisation, providing a good comparison data set.

### 2.3 ASAP-MS measurement

The ASAP-MS measurements were conducted on an Advion express**ion**® compact mass spectrometer-S (CMS-S) ASAP-MS instrument (Advion Ltd, Harlow, United Kingdom) [47] using the protocol developed and described previously [7]. The glass capillaries used for sample loading (Advion ASAP S01 short) were sterilised by heating in an oven for 30 min at 250 °C, and were then stored in a dessicator.

To make a measurement, a clean capillary was loaded into the ASAP probe and the probe was inserted into the spectrometer ion source to record a background measurement for 30 s under ‘high temperature low fragmentation’ ion source conditions. The probe was then removed from the ion source, and the tip cooled with a small amount of methanol and dried with lens tissue (Thorlabs MC-5 lens tissue) before loading the sample. Prior to measurement, each plasma sample was thawed at room temperature and then mixed by vortex mixer for 15 s to obtain a homogeneous sample. A small amount of sample was transferred to the probe tip by sampling from the internal surface of the sample tube just above the level of the plasma surface. The probe was then inserted into the ASAP-MS instrument and data acquired for 30 s, before the probe was removed, cleaned with methanol, and wiped dry with lens tissue. The sampling, measurement, and cleaning steps were repeated four more times using the same capillary for a total of five measurements per sample. The capillary was then discarded and a fresh capillary was inserted for the next sample [7].

### 2.4 Data processing

The raw data files were exported for analysis via the Advion Data Express software and processed using a Python 3.7 script [48]. Each raw data file contains a large number of mass spectra recorded as a function of time during a data run. The Python script extracted the portions of data recorded when the ASAP probe was present in the ion source, averaged the data over each 30 s acquisition, performed a background subtraction, and binned the resulting spectra to unit mass. This resulted in a set of 990 features (i.e. *m*/*z* intensities) per spectrum for use in the data analysis described in the following section. No further feature reduction was implemented. For the OxAMI samples, the ten background-corrected spectra recorded for each sample were then averaged to generate a single mass spectrum for each sample, to which the various normalisation methods were applied. For the very small set of OxAAA samples, this final averaging step was not performed, and all ten spectra recorded for each sample were used in training the ML algorithms (i.e. we employed 20 patient samples and 200 spectra for each of the ‘healthy volunteer’ and ‘small aneurysm’ classes). Care was taken during the training process to ensure that spectra recorded for any particular patient were never split across training and test data partitions, i.e. either all ten spectra were included in the training data partition, or all ten spectra were included in the test partition.

### 2.5 Method evaluation

The aim of this study was to determine how the normalisation, scaling, and transformation methods employed during pre-processing of ASAP mass spectra affected the outcomes of machine-learning classification. The effects were quantified using two separate approaches: clustering efficiency evaluation; and a machine learning method.

**Fig. 3:**
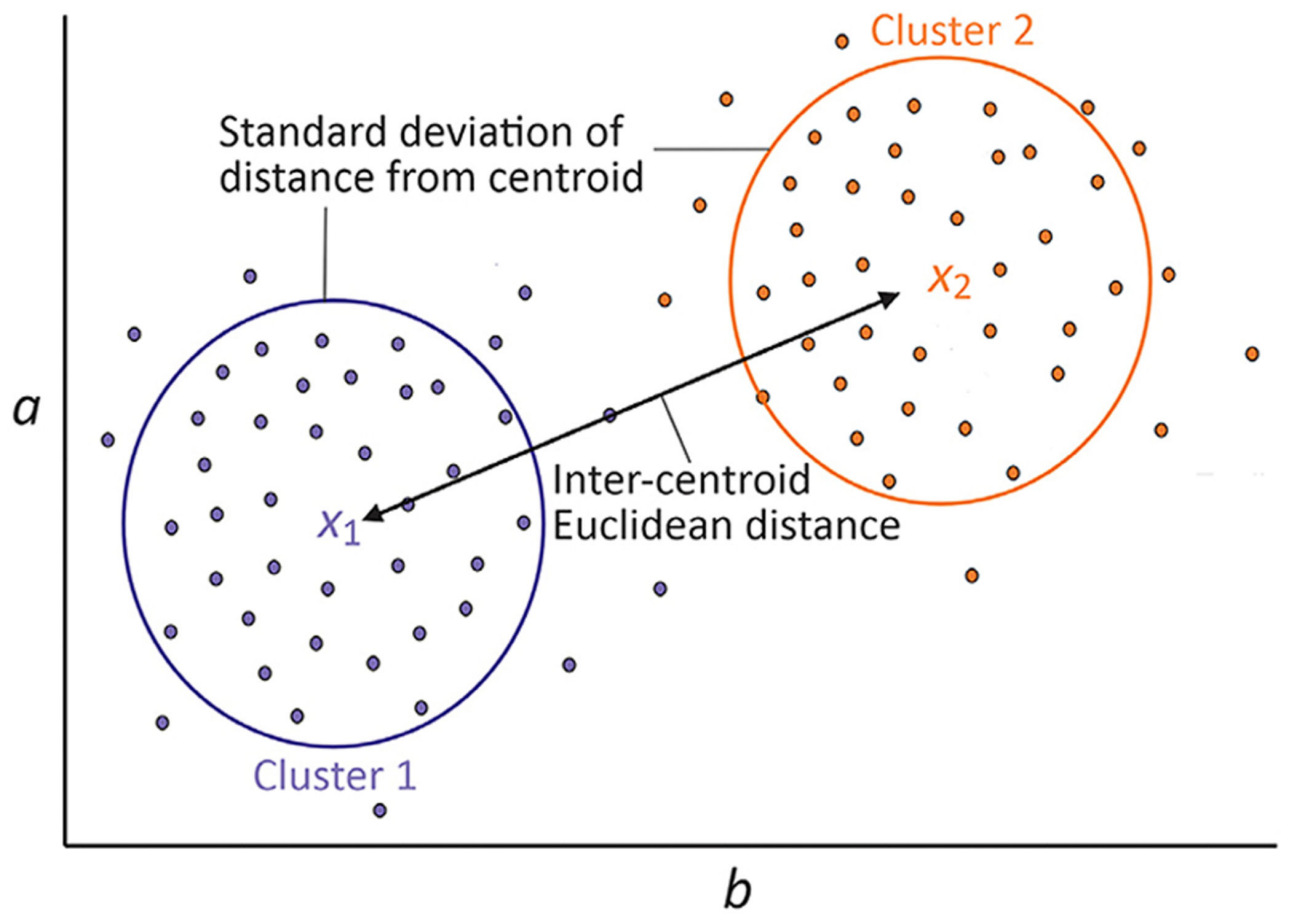
A two-dimensional graphical representation of the cluster ratio analysis. Data are shown plotted with respect to axes *a* and *b*, which represent two *m*/*z* values. The centroids of the clusters for the two classes are labelled *x*_1_ and *x*_2_, and the cluster sizes and cluster separation are shown.

**Cluster efficiency evaluation:** The extent of clustering of the mass spectra into the two categories of interest (as defined in Section 2.2) was assessed by calculating the cluster size to centroid separation ratio (‘Clustering ratio’), *C*_*R*_. Each mass spectrum is treated as a vector of *m*/*z* intensities, and labelled according to which category it belongs. The centroid for each cluster is simply the average of the vectors within that cluster. The centroid separation is defined as the Euclidean distance between the two centroids, and the cluster size is defined as the standard deviation of the Euclidean distances of each mass spectrum in the cluster from the cluster centroid. The centroid separation ratio is then the centroid separation divided by the sum of the two cluster sizes. A large *C*_*R*_ value represents a data set within which individuals within the same group are tightly clustered together, and there is a clear separation between the two clusters of the two data groups. A graphical representation is shown in two dimensions in Fig. 3.

**Machine learning evaluation:** The various normalisation, scaling and transformation methods described were also assessed based on the extent of successful classification via five different supervised classification models. The models used were K-nearest neighbours (KNN) [49], support vector machines (SVM) [50], linear discriminant analysis (LDA) [51], naive Bayes classifier (NBC) [51], and random forest classifier (RFC) [52], spanning a range of different approaches to classification. ML analysis was conducted in MATLAB 2022 A, and hyperparameters were optimised using the automated hyperparameter optimisation function. For the OxAMI data, all methods were applied within a cross-validation protocol employing 50 different 80:20 partitions of the data into training and test data sets. The predictive accuracy was obtained from the summed confusion matrix across these partitions. Repeating the analysis across multiple data partitions prevents overfitting arising from a particular selection of patients for the training groups. For the AAA data set, we used a hold-*n*-out method to ensure that samples from the same patient could not exist within both test and training partitions, in order to avoid data leakage.

The predictive accuracies of the ML models were assessed using Cohen’s *κ* statistic:

(17) $$\kappa =\frac{P_{0}-P_{e}}{1-P_{e}}=\frac{2\times (TP\times TN-FN\times FP)}{\left(TP+FP)\times (FP+TN)+(TP+FN)\times (FN+TN\right)}$$

The first definition in Eq. (17) compares the predictive accuracy *P*_0_ of the model with the probability *P*_*e*_ of a correct prediction based purely on chance. The second definition formulates *κ* in terms of the fraction of true positive (TP), true negative (TN), false positive (FP) and false negative (FN) predictions [53]. The *κ* scores were interpreted using the levels of agreement (weak, fair, moderate, strong, and very strong) as defined by McHugh [53]. The reported values for *κ* and uncertainties *Δκ* are the mean and standard deviation of the *κ* scores obtained over the 50 data partitions.

## 3 Results and discussion

### 3.1 The effect of normalisation, scaling and transformation methods on clustering efficiency

**Fig. 4:**
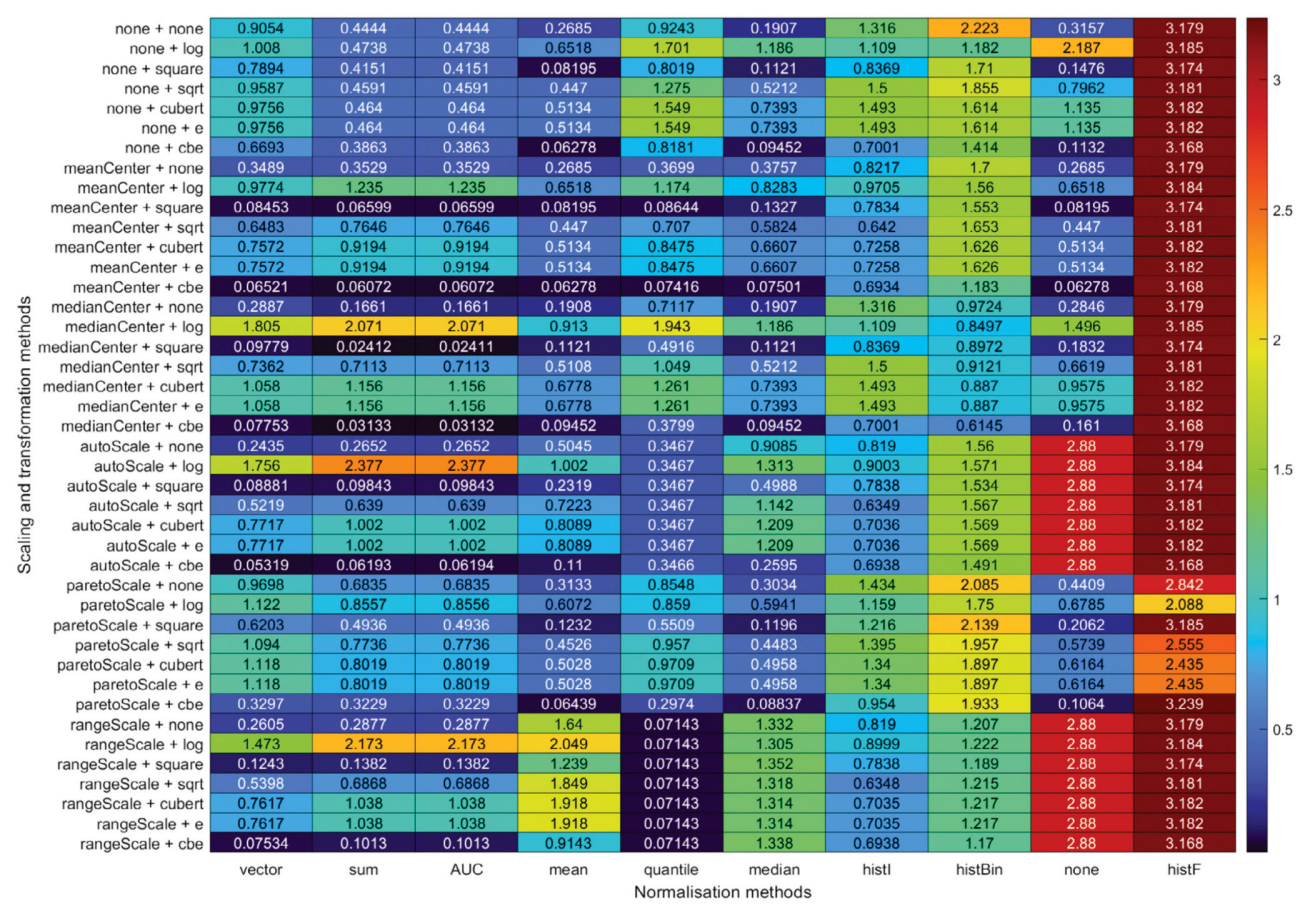
Clustering ratios obtained using each of the normalisation, scaling, and transformation methods for the OxAAA data clustered into ‘healthy volunteer’ and ‘small aneurysm’ patient groups. The colour map represents the *C*_*R*_ value from low (blue) to red (high).

As explained in Section 2.5, the mass spectra recorded for plasma samples from patients and volunteers enrolled in the OxAAA study were assessed for the extent of clustering, defined by the clustering ratio *C*_R_, into ‘healthy volunteer’ and ‘small aneurysm’ groups after each of the normalisation methods had been applied. The colour map table in Fig. 4 shows the full set of *C*_R_ scores for all of the combined methods. The maximum *C*_R_ value was obtained using the histogram function normalisation method, combined with pareto scaling and a cubic transform. The histogram function method generated high *C*_R_ values with all combinations of scaling and transformation methods. When employing no normalisation method, both range scaling and autoscaling also performed well with all combinations of transform method.

**Fig. 5:**
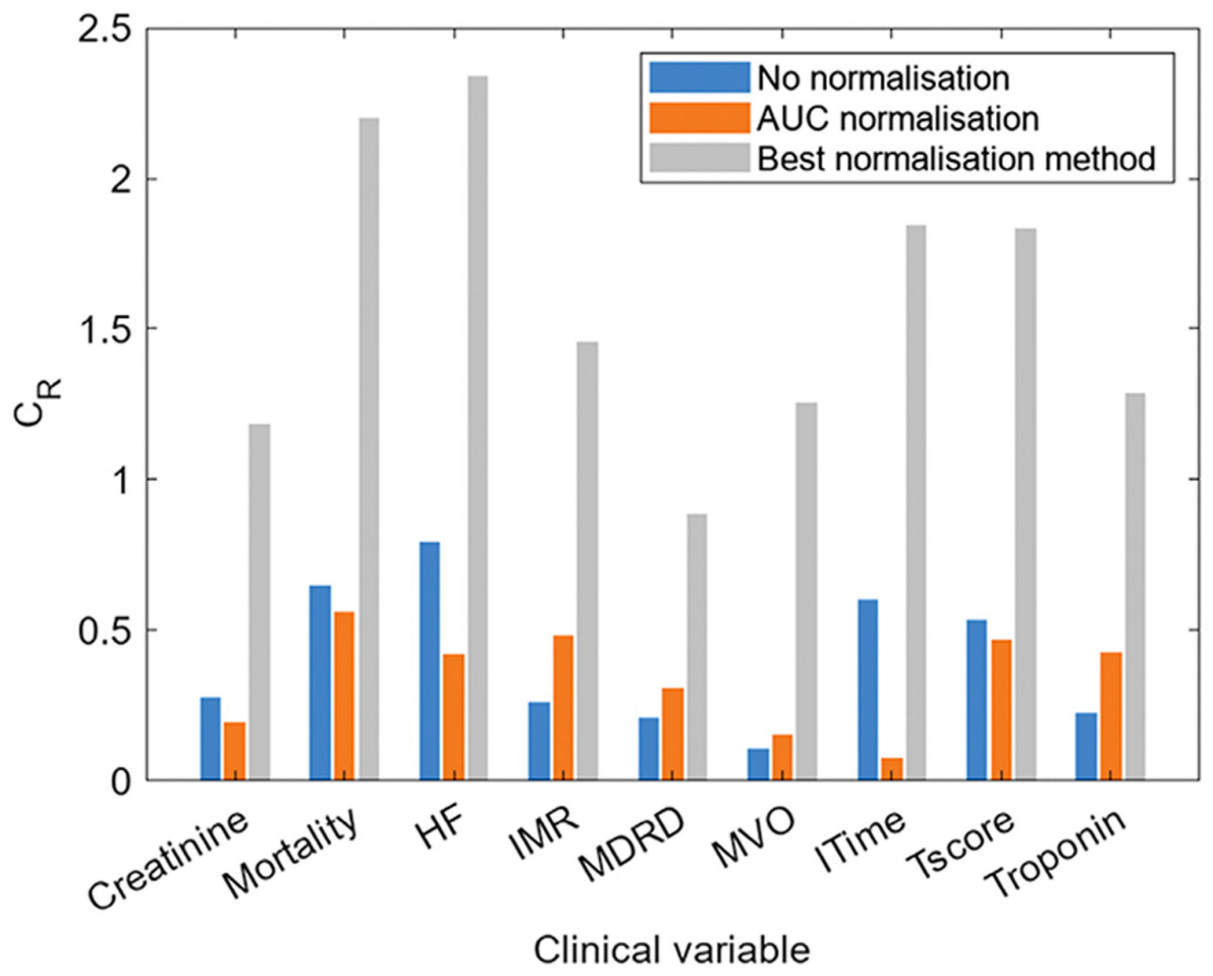
Comparison between the clustering ratio obtained using the best-performing normalisation, scaling and transform method (grey) for the OxAMI clinical variables and the clustering ratio obtained with no normalisation (blue) and AUC normalisation (orange).

The mass spectra recorded for patients enrolled in the OxAMI study were used for nine different classifications, based on the nine clinical variables and associated categories described in Section 2.2. The methods that gave the highest *C*_R_ values for each clinical variable are shown in Table 3. The most common high-performing combination of methods was found to be Histogram bin normalisation, no transformation, and no scaling. A comparison between no methods, the previously used AUC method, and the best-performing method from the present analysis, is shown for each clinical variable in Fig. 5. In all cases, clustering was greatly improved in the best performing method.

### 3.2 The effect of normalisation, scaling and transformation methods on machine learning classification

**Fig. 6:**
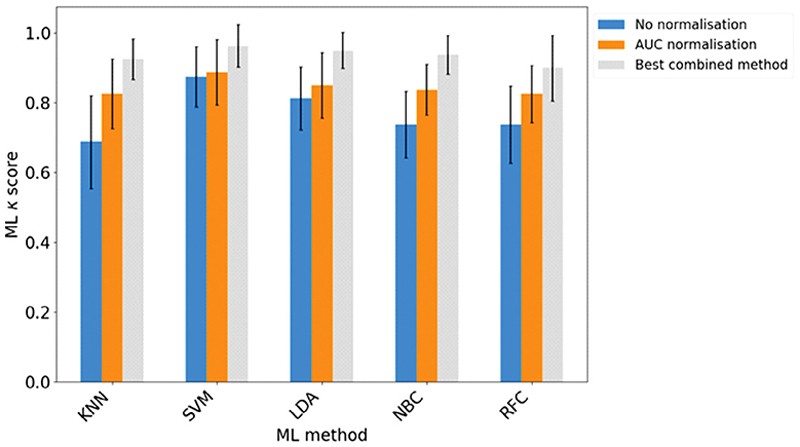
*κ* scores obtained for each of the five ML algorithms used to classify the OxAAA data into ‘healthy volunteer’ and ‘small aneurysm’ categories, employing either no normalisation (blue), AUC normalisation (orange), or the best performing normalisation, scaling, and transformation method (grey). Error bars show the standard deviation in *κ* over 50 data partitions.

Fig. 6 shows the *κ* scores obtained for each of the five ML algorithms used to classify the OxAAA data into ‘healthy volunteer’ and ‘small aneurysm’ categories. The figure shows the results obtained with no data normalisation, with area-under-the-curve (AUC) normalisation, and using the combined normalisation, scaling, and transformation method that led to the best performance of the relevant ML algorithm. The best-performing methods for each ML algorithm are listed in Table 4.

The results show that AUC normalisation leads to better classification than no normalisation, and that optimisation of the normalisation, scaling, and transformation methods leads to further improvement in the classification performance of all ML models. With this optimisation, the RFC classifier had the lowest (but still very good) accuracy of 93% (*κ* 0.9), while all other ML methods yielded accuracies of 96% (*κ* 0.925) or higher. No clear ‘winner’ emerges as a combination yielding the best performance over multiple ML methods, though we note that there is more variation amongst the best-performing transformation methods than the best-performing normalisation and scaling methods.

**Fig. 7:**
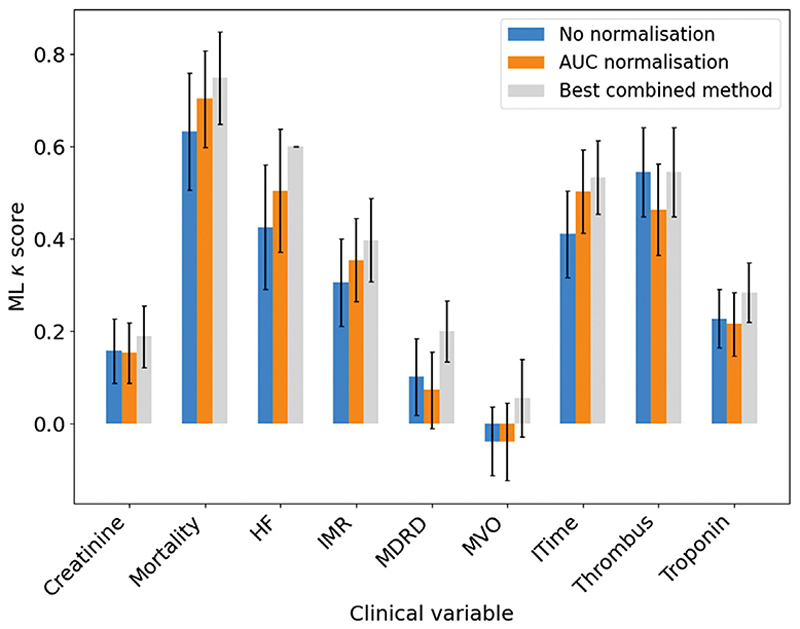
Comparison between the *κ* scores obtained for the LDA classification using the best-performing normalisation, scaling and transform method (grey) for the OxAMI clinical variables, and those obtained with no normalisation (blue) and AUC normalisation (orange). Error bars show the standard deviation in *κ* over 50 data partitions.

Moving on to consider the OxAMI data, Fig. 7 shows the *κ* scores obtained for classifications into the various patient groups described in Table 2 performed using the LDA algorithm, which was found to give reproducible and accurate predictions in previous analyses. We note that LDA uses Bayes’ Theorem to classify data, and therefore assumes normally distributed and homoscedastic data classes. We also note that this approximation is likely to improve in correlation with increasing cluster ratio *C*_*R*_. Having said this, the other four ML algorithms investigated yielded very similar results, which can be found in the Supporting Information. As for the OxAAA data shown in Fig. 6, we show the *κ* scores obtained with no data normalisation, with area-under-the-curve (AUC) normalisation, and using the combined normalisation, scaling, and transformation method that led to the best performance of the ML algorithm. The best-performing methods for each clinical variable are listed in Table 5.

Improvements in predictive accuracy of the LDA model were obtained for each classification when the normalisation/scaling/transformation method was optimised, with the greatest improvements observed for the heart failure diagnosis and MVO classifications. Normalisation methods that performed well were mean, median, vector, and AUC normalisation. The scaling methods that performed well were mean centre scaling, median centre scaling, and auto scaling. Many transformation methods were found to be effective, but were highly data-set specific. Unlike for the clustering evaluation method, no single combination of methods consistently performed best. Perhaps surprisingly, there was found to be little or no correlation between the methods that were determined in Section 3.1 to result in a high clustering efficiency, such as the histogram-based methods, and those that yielded the highest ML classification accuracy. This lack of correlation is shown graphically in Figure S2 of the Supporting Information, using examples from both the OxAAA and OxAMI data analysis.

As was the case for the OxAAA data, the increases in *κ* score when going from ‘no normalisation’ to the best performing method employed for the OxAMI data are modest, ranging from 0.1–0.2 in most cases. Such increases generally lie within a single *κ* interpretation band, as described by McHugh [53]. Improvements were not universal: for thrombus score, ‘no normalisation’ yielded the highest accuracy model. The impact of normalisation methods on the outcome of ML-based classification, at least for the methods investigated in the present study, can therefore be said to be relatively low.

## 4 Conclusions

We have investigated the effects on subsequent classification analysis of the choice of normalisation, scaling, and transformation methods used when processing ASAP-MS mass spectra of blood plasma. We have not included methods relying on internal or external standards within our study, as such methods are not well suited to our applications. However, we note that additional normalisation options become available in cases where such standards can be used.

We have found that the choice of method has a substantial effect on clustering of the data into different patient cohorts, but a much smaller effect on machine-learning-based classification. Histogram-derived methods, particularly when combined with Pareto scaling or cubic transformations, enhanced cluster separability for both the OxAAA and OxAMI data sets employed within this study, while more conventional methods (mean, median, vector, and AUC normalisation) yielded the best ML classification performance across multiple algorithms. Importantly, we found that high clustering ratio scores do not necessarily correlate with improved supervised classification outcomes, underscoring the need to consider subsequent data analysis methodology carefully when optimising data preprocessing pipelines.

We note that the optimal normalisation methods identified in this study for ASAP-MS measurements on human plasma may or may not generalise across other instruments or biological sample types. While it is probably not feasible for researchers to conduct a comprehensive evaluation of normalisation methods in each case, we can draw a few general conclusions from our work:

1. For high quality data, the specific normalisation approach chosen is unlikely to influence the overall analytical outcome substantially, particularly when employing standard machine learning models. Based on our own experience, time is far better spent on optimising measurement protocols to obtain good quality measurements than on trying to optimise normalisation processes on poor quality data.
2. In general, commonly used approaches such as sum normalisation or unit area under the curve (unit AUC) are likely to perform adequately and provide a reliable estimate of classification accuracy. Although modest improvements in accuracy may be achievable through alternative normalisation techniques, these gains are typically small. Researchers should therefore consider the cost-benefit relationship between the additional effort required in order to carry out an extensive normalisation study and the magnitude of any potential performance improvement.
3. For studies seeking to maximise sensitivity with a clear analytical outcome to test against, it can be valuable to carry out a systematic evaluation of a range of normalisation approaches. The goal is then to identify which methods are most effective *given the characteristics of the data set and the objectives of the data analysis*.
4. Employing data transformation methods in conjunction with an appropriate normalisation protocol often leads to improved analytical performance and more robust classification outcomes, so this approach may be worth exploring in new applications.

## Supplementary Material

Supplementary material related to this article can be found online at https://doi.org/10.1016/j.ijms.2025.117553.

Supporting Information

## Figures and Tables

**Table 1:** Normalisation, scaling and transformation methods used in the present study.

Normalisation	Scaling	Transformation
None	None	None
AUC	Mean centre scaling	Log
Mean	Median centre scaling	Exponential
Median	Auto scaling	Square
Quantile	Pareto scaling	Square root
Vector	Range scaling	Cube
Minimised vector		Cube root
Histogram bin position		
Histogram intensity		
Histogram function		

**Table 2:** Categories used for the binary classification of mass spectra according to the clinical variables of interest in the OxAMI study.

Clinical parameter	Classification conditions	Samples, *n*	Samples/category, *n*/2
MVO	0 or > 0	128	64
TScore	1-3 or 4–5	90	45
ITime	< 6 hours or > 6 hours	92	46
IMR	< 40 or > 40	126	63
HFD	No or Yes	50	25
Patient mortality	No or Yes	46	23
Peak troponin	< 50 or > 50	220	110
Creatinine on admission	< 75 or > 75	242	121

**Table 3:** Best-performing normalisation, scaling and transform methods for OxAMI variables assessed by clustering ratio.

Clinical variable	Normalisation	Transform	Scaling
Creatinine	Histogram bin	None	None
Mortality	Histogram function	log	Median centre
HF	AUC	log	Auto
IMR	Histogram bin	None	None
MDRD	Histogram intensity	cube	Range scaling
MVO	Histogram bin	None	None
ITime	Histogram bin	None	None
TScore	Histogram bin	None	None
Troponin	Histogram bin	None	None

**Table 4:** The best-performing normalisation, scaling, and transformation methods for ML classification of OxAAA spectra into ‘healthy volunteer’ and ‘small aneurysm’ categories. These methods correspond to the *κ* scores plotted as grey bars in Fig. 6.*Δκ* is the one-standard-deviation uncertainty in the *κ* score determined over 50 partitionings of the data into training and test sets.

ML method	Normalisation	Scaling	Transformation	*κ*	*Δk*	Accuracy
KNN	None	None	Cube root	0.925	0.059	96%
SVM	AUC	None	Square root	0.963	0.061	98%
LDA	AUC	Mean centre	Cube	0.950	0.051	98%
NBC	Quantile	None	Exp	0.938	0.056	97%
RFC	AUC	None	Square	0.900	0.094	93%

**Table 5:** Best-performing normalisation, scaling and transform methods for OXAMI variables assessed by LDA ML classification accuracy.

Clinical variable	Normalisation	Scaling	Transformation	% Accuracy range
Creatinine	Mean	None	Log	<60%
Mortality	Vector	Mean centre	None	80-90%
HF	Min vector	Mean centre	Log	80-90%
IMR	AUC	Mean centre	Square-root	70-80%
MDRD	Median	Median centre	Cube-root	60-70%
MVO	Mean	Auto	Cube-root	<60%
ITime	Vector	Mean centre	None	70-80%
TScore	None	None	None	70-80%
Troponin	Median	None	Cube-root	60-70%

## Data Availability

Data will be made available on request.
