# Supplementary material for: Optimising the choice of normalisation method for use in machine-learning classification of human blood plasma ambient ionisation mass spectra
Source: Int J Mass Spectrom. Author manuscript; Available in PMC 2026 Feb 23. (PMC7618781; doi:10.1016/j.ijms.2025.117553)
Supplement: Supporting Information [file EMS212372-supplement-Supporting_Information.pdf]

# Supporting information: Optimising the choice of normalisation method for use in machine-learning classification of human blood plasma ambient ionisation mass spectra

Annabel S. J. Eardley-Brunt[1], Liwen Song[1], The Oxford Acute Myocardial Infarction (OxAMI) Study[2], The Oxford Abdominal Aortic Aneurysm (OxAAA) Study[3], and Claire Vallance[1]

## 1 Effect of normalisation method on machine learning classification for OxAMI samples: other machine learning methods

In the main manuscript we presented results for classification of the OxAMI data using the LDA machine learning algorithm. As explained in the Methods, we also used four other supervised ML classification models. These yielded similar results to the LDA model, which are presented here.

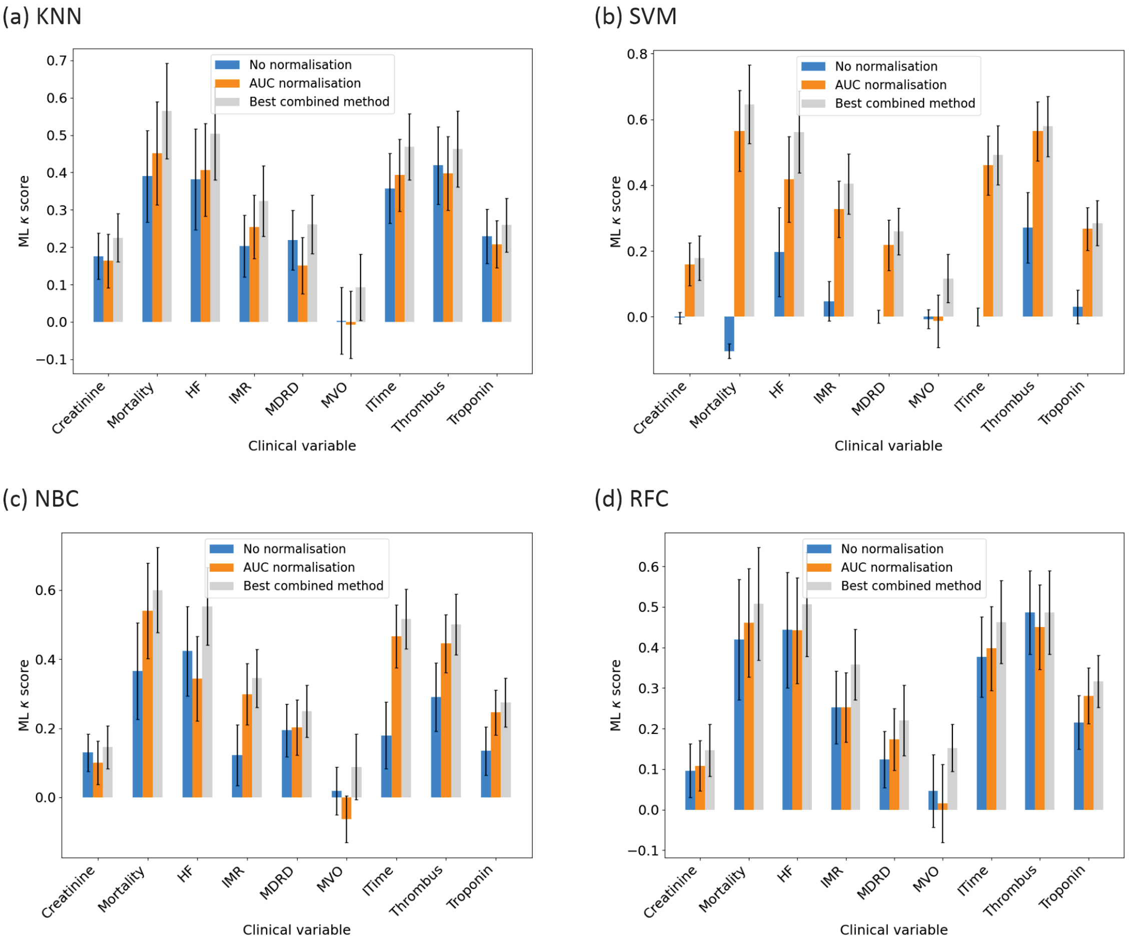


Figure 1: Comparison between normalisation, scaling and transform methods for ML classification according to OXAMI clinical variables evaluated by $\kappa$ score. The $\kappa$ score is plotted for (a) KNN, (b) SVM, (c) NBC, and (d) RFC models when no normalisation, scaling and transformation is used (blue); when AUC normalisation is used with no other scaling or transformations (orange); and when the normalisation, scaling and transformation combined method that results in the best ML model performance is used (grey). Error bars show the standard deviation in $\kappa$ over 50 partitions.

The classification results when employing (i) no normalisation, (ii) AUC normalisation, and (iii) the combined normalisation, scaling, transformation methods that yielded the best classification performance are shown here for the K nearest neighbours (KNN), support vector machines (SVM) modelm, Naive Bayes classifier (NBC), and random forest classifier (RFC) models in Figure S1.

The corresponding normalisation methods are detailed in Tables S1 to S4. The results when employing different ML models are very similar, with the exception of the SVM model, which performed very poorly when no normalisation was employed.

Table 1: Best normalisation, scaling and transform methods for OXAMI variables assessed by KNN ML classification accuracy

| | KNN | | |
|---|---|---|---|
| | Normalisation | Scaling | Transformation |
| Creatinine | Vector | None | Cube-root |
| Death | Vector | Mean centre | None |
| HF | AUC | Median centre | Log |
| IMR | Vector | None | Cube |
| MDRD | Vector | None | Cube |
| MVO | None | Mean centre | Log |
| Pain | None | None | Cube-root |
| Tscore | Quantile | None | Exp |
| Troponin | Quantile | None | Square |

Table 2: Best normalisation, scaling and transform methods for OXAMI variables assessed by SVM ML classification accuracy

| | SVM | | |
|---|---|---|---|
| | Normalisation | Scaling | Transformation |
| Creatinine | mean | Mean centre | Exp |
| Death | None | None | Square-root |
| HF | AUC | Median centre | Log |
| IMR | Sum | Mean centre | None |
| MDRD | Vector | None | Square |
| MVO | Quantile | Range | Square |
| Pain | Sum | None | Log |
| Tscore | Sum | None | None |
| Troponin | Sum | None | Cube |

Table 3: Best normalisation, scaling and transform methods for OXAMI variables assessed by NBC ML classification accuracy

| | NBC | | |
|---|---|---|---|
| | Normalisation | Scaling | Transformation |
| Creatinine | None | None | Log |
| Death | Quantile | None | Square-root |
| HF | mean | None | Log |
| IMR | Vector | None | Log |
| MDRD | Vector | None | Cube |
| MVO | AUC | Median centre | Cube |
| Pain | AUC | Median centre | none |
| Tscore | Quantile | Median centre | Cube-root |
| Troponin | Sum | None | None |

Table 4: Best normalisation, scaling and transform methods for OXAMI variables assessed by RFC ML classification accuracy

| | RFC | | |
|---|---|---|---|
| | Normalisation | Scaling | Transformation |
| Creatinine | Vector | Median centre | Cube |
| Death | AUC | Median centre | None |
| HF | Mean | pareto | Square-root |
| IMR | Vector | Median centre | Exp |
| MDRD | AUC | Mean centre | None |
| MVO | Min-vector | Auto | None |
| Pain | Sum | Median centre | Log |
| Tscore | None | None | None |
| Troponin | Vector | Median centre | Cube |

# 2 Comparison between clustering performance and ML classification performance in relation to optimisation of normalisation approach

In the main manuscript we considered the effect of normalisation approach on clustering of the data into categories, measured by the clustering ratio $C_{\mathrm{R}}$, and on ML-based classification of the data into categories using a variety of ML algorithms. We noted that there appears to be little correlation between the normalisation methods that result in the highest $C_{\mathrm{R}}$ and the methods that yield the best-performing ML classification. We provide evidence for this in the following by showing selected examples.

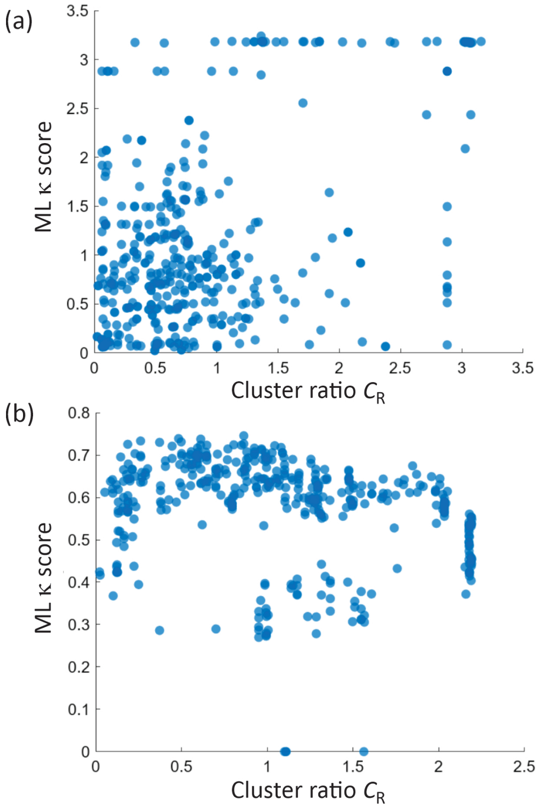


Figure 2: Comparison of normalisation evaluation methods for (a) OxAAA data classified into 'healthy volunteer' and 'small aneurysm' patient groups; and (b) OxAMI data classified by patient mortality (yes or no). The cluster ratio is plotted against ML $\kappa$ score. No correlation was observed in either case.

Figure S2 contains scatter plots showing the lack of correlation between $\kappa$ score obtained in the ML classification and the clustering ratio $C_R$ obtained in the clustering analysis. The cases

shown are: (a) classification of OxAAA data into samples corresponding to healthy volunteers and patients with small aneurysms, and (b) classification of OxAMI data according to patient mortality (yes or no). The lack of correlation suggests that the location of a data point in vector space does not in itself define the success of the ML classification for the data sets considered. Good ML classification is often observed even when the data are not particularly well clustered into the relevant categories in vector space. This perhaps indicates (unsurprisingly) that the ML classification focuses on particular features (i.e. dimensions) of the data, rather than on the complete vector space. This is corroborated by the success of ML classification analysis performed on reduced data sets obtained via feature reduction, which selects a subset of $m/z$ peaks that have statistically different intensities correlating to the categories of interest.

# 3 Sensitivity analysis

A Monte Carlo power permutation test was conducted across each of the data sets used to build classification models in order to determine the statistical power of each classification as a function of the number $n$ of patients included ($\alpha$ = 0.05, 50 repeats, CV folds = 5, 100 permutations)[1, 2, 3, 4, 5, 6] The analytical endpoint used was an SVM classifier and $\kappa$ score, as used in the classification analysis. This method was used as the number of variables ($m/z$ peaks) involved in each mass spectrum, and the high degree of covariance between these peaks, may lead to some of the more standard power calculations being inappropriate. Table 3 shows that the number of samples required to reach the commonly used threshold of 0.8 was lower than the number of samples used in most cases, with a small number of exceptions for the smallest datasets[7]. It was observed that the AAA dataset had lower sample number requirements when using the patient-averaged data than when using the non-averaged data (i.e. the individual spectral repeats), most likely as a result of the better signal-to-noise ratio achieved when a number of individual repeat spectra are averaged.

Table 5: Permutation power analysis results for the data sets tested, showing the number of samples used vs the number of samples required to achieve a power of 0.8. AAA = abdominal aortic aneurysm; HV = healthy volunteer; IMR = index of microcirculatory resistance; MVO = microvascular obstruction.

| Clinical variable being analysed | N used in analysis | N samples required to achieve power of 0.8 |
|---|---|---|
| Small AAA vs HV (averaged spectra for each patient) | 20[1] | 10 |
| Small AAA vs HV (individual spectra) | 200 | 25 |
| Creatine | 121 | 75 |
| Mortality | 23 | 25 |
| Heart failure diagnosis | 25 | 25 |
| IMR | 63 | 65 |
| MVO | 64 | 35 |
| Ischaemic time | 46 | 40 |
| Thrombus score | 45 | 50 |
| Troponin | 110 | 80 |

## 3.1 Sensitivity testing for the patient-averaged and non-averaged AAA data sets

In addition to the tests described above, a univariate 2-sample $t$-test was conducted across both the patient-averaged and non-averaged AAA data for each $m/z$ peak [8]. The mean —$t$— values for the averaged and non-averaged AAA data were 0.773 and 1.340, respectively. The distribution of $t$ statistics is shown in Figure 3. The average $t$ statistic is found to be higher for the non-averaged data, suggesting a higher power than the averaged data. Usually it might be expected that the aggregated data would have a higher stability and less noise, and so would have higher $t$ scores. However, in this case the non-aggregated data adds power by pseudo-replication. The power analysis showed that a very low number of samples $n$ was needed to reach the power level typically used for medical data. Further analysis (not included in the present publication) has shown that there is no difference in the ML outcomes obtained using the two data sets.

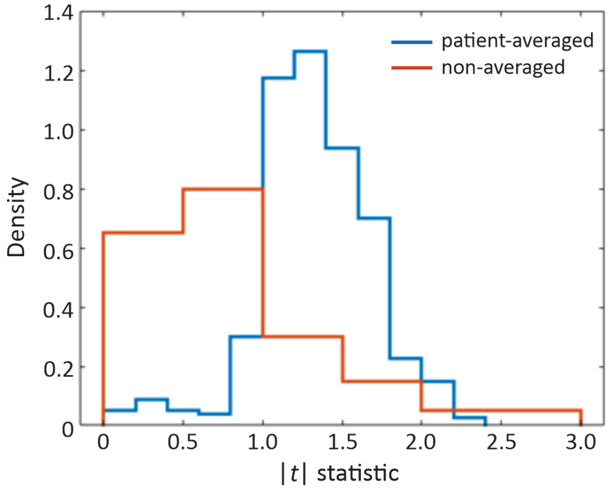


Figure 3: Histogram plot of the distribution of absolute $t$ statistic across all of the $m/z$ features when using patient-averaged (orange) and non-averaged (blue) AAA data sets.

# References


[1] Fi MO, Garriga GC. Permutation Tests for Studying Classifier Performance. Journal of Machine Learning Research. 2010;11:1833-63.

[2] Holland B. Monte Carlo simulation of the power of a test for the exponential distribution of survival times. Computer Methods and Programs in Biomedicine. 1989;29:245-50.

[3] Graebner RW, Inc PRA. Study Design with SAS: Estimating Power with Monte Carlo Methods – SUGI24 Conference Poster; 1999.

[4] Monte Carlo Power Analysis for Indirect Effects;. Https://schoemanna.shinyapps.io/mc_power_med/.

[5] GraphPad Prism 10 Statistics Guide - Monte Carlo example: Power of unpaired t test;. Https://www.graphpad.com/guides/prism/latest/statistics/stat_example_power_of_unpaired_t_te.htm.

[6] Zeleny D. Monte Carlo permutation test in constrained ordination; 2025.

[7] Serdar CC, Cihan M, Yücel D, Serdar MA. Sample size, power and effect size revisited: simplified and practical approaches in pre-clinical, clinical and laboratory studies. Biochemia Medica. 2020;31:010502.

[8] UCLA. Power analysis for two-group independent sample t-test: G*Power Data Analysis Examples; 2024. Https://stats.oarc.ucla.edu/other/gpower/power-analysis-for-two-group-independent-sample-t-test/.
